# Historical Datasets Support Genomic Selection Models for the Prediction of Cotton Fiber Quality Phenotypes Across Multiple Environments

**DOI:** 10.1534/g3.118.200140

**Published:** 2018-03-20

**Authors:** Washington Gapare, Shiming Liu, Warren Conaty, Qian-Hao Zhu, Vanessa Gillespie, Danny Llewellyn, Warwick Stiller, Iain Wilson

**Affiliations:** * CSIRO Agriculture & Food, GPO Box 1600, Canberra, ACT 2601, Australia; † CSIRO Agriculture & Food, Locked Bag 59, Narrabri, NSW 2390, Australia

**Keywords:** Genomic prediction, Genomic selection, Bayesian models, marker × environment interaction, *Gossypium hirsutum*, GenPred, Shared Data Resources

## Abstract

Genomic selection (GS) has successfully been used in plant breeding to improve selection efficiency and reduce breeding time and cost. However, there has not been a study to evaluate GS prediction models that may be used for predicting cotton breeding lines across multiple environments. In this study, we evaluated the performance of Bayes Ridge Regression, BayesA, BayesB, BayesC and Reproducing Kernel Hilbert Spaces regression models. We then extended the single-site GS model to accommodate genotype × environment interaction (G×E) in order to assess the merits of multi- over single-environment models in a practical breeding and selection context in cotton, a crop for which this has not previously been evaluated. Our study was based on a population of 215 upland cotton (*Gossypium hirsutum*) breeding lines which were evaluated for fiber length and strength at multiple locations in Australia and genotyped with 13,330 single nucleotide polymorphic (SNP) markers. BayesB, which assumes unique variance for each marker and a proportion of markers to have large effects, while most other markers have zero effect, was the preferred model. GS accuracy for fiber length based on a single-site model varied across sites, ranging from 0.27 to 0.77 (mean = 0.38), while that of fiber strength ranged from 0.19 to 0.58 (mean = 0.35) using randomly selected sub-populations as the training population. Prediction accuracies from the M×E model were higher than those for single-site and across-site models, with an average accuracy of 0.71 and 0.59 for fiber length and strength, respectively. The use of the M×E model could therefore identify which breeding lines have effects that are stable across environments and which ones are responsible for G×E and so reduce the amount of phenotypic screening required in cotton breeding programs to identify adaptable genotypes.

The main goal of commercial crop breeding programs is to identify the best performing individuals (or genotypes) as potential cultivars, with the secondary goal to identify genotypes that can be used as parents in future crosses to advance specific breeding objectives. However, the principal limitation in most crop breeding programs is the long timeframes required for the completion of one cycle of breeding, testing and selection, often in the order of 10 years. Genomic selection (GS) emerged from the need to improve prediction of complex traits and to reduce selection cycles and phenotyping (Meuwissen *et al.* 2001). In GS, a training population is phenotyped and genotyped to train a model, which can be used to predict genomic estimated breeding values (GEBV) of another set of individuals that has only been genotyped but not phenotyped (Meuwissen *et al.* 2001). Under a GS model, precision-phenotyping is most important when evaluating a training population because that dataset provides the basis for developing the statistical model that is then used to predict phenotypic performance in related members of a breeding population (Cobb *et al.* 2013). Likewise, a proper phenotypic analysis and appropriate modeling are crucial prerequisites for accurate calibration of genomic prediction procedures (Bernal-Vasquez *et al.* 2014; 2017).

The potential utility of GS in crop improvement is currently being intensively studied in different types of plant populations and traits (Heffner *et al.* 2011; Heslot *et al.* 2015), but has been most successfully used in dairy cattle breeding (Goddard and Hayes 2009; Hayes *et al.* 2009). Initial studies in plants have included data on several important traits for major crops such as grain amylase activity in barley (Lorenzana and Bernardo 2009), grain moisture and grain yield in maize (Zhao *et al.* 2012), grain yield and plant height in rice (Spindel *et al.* 2015), grain yield, resistance to *Fusarium* head blight and stem rust resistance in wheat (Crossa *et al.* 2014; Rutkoski *et al.* 2014; Huang *et al.* 2016) and stem diameter, pulp yield, and rust resistance in eucalypts (Grattapaglia and Resende 2011). The body of plant GS research has grown substantially since the first early descriptions of using genomic selection to predict unmeasured phenotypes. However, we still lack sufficient understanding of the potential value of GS in an operational breeding program, particularly for polygenic traits of agronomic significance, and the factors that might determine its success in specific crop species.

The accuracy of GS is crucial for its successful application in a practical breeding scheme. An increasing number of studies have provided an empirical estimation of genomic prediction accuracies for different traits and crops such as, barley, maize, and wheat and trees like eucalypts (Lin *et al.* 2014), and shown that they can vary greatly depending on whether the crops is inbred or outbred. In fact, determining in advance the prediction accuracy for a specific population and a specific trait is difficult, because accuracy is influenced by many factors (Desta and Ortiz 2014) such as the marker density relative to the effective population size, the linkage disequilibrium between markers and quantitative trait loci (QTL), the suitability of the prediction model with regards to the population genetics (relationship between individuals in the population, genetic structure), and the architecture of the trait of interest (*e.g.*, heritability, underlying gene actions) (Goddard 2009; Heslot *et al.* 2013b; Jarquín *et al.* 2014; Isidro *et al.* 2015).

Since Meuwissen *et al.* (2001) first proposed this concept of GS along with several models, numerous statistical methods, including parametric and nonparametric methods, have been used to predict quantitative traits. Parametric methods include best linear unbiased prediction (BLUP; Henderson 1975), least absolute shrinkage and selection operator (LASSO; Tibshirani 1996), partial least squares (PLS; Geladi and Kowalski 1986) and Bayesian based methods such as Bayes Ridge Regression (BRR), BayesA, BayesB and Bayesian LASSO (Yi and Xu 2008); nonparametric methods include random forests (Svetnik *et al.* 2003) and Reproducing Kernel Hilbert Spaces regression (RKHS) (de los Campos *et al.* 2009; de los Campos *et al.* 2010). Recently, many investigators have evaluated the performance of various statistical methods used in GS. de los Campos *et al.* (2013) gave an overview of the parametric methods and concluded that BLUP performs well for most traits and BayesB yields slightly higher predictive accuracy for traits with large-effect quantitative trait loci (QTL). Riedelsheimer *et al.* (2012a,b) compared the predictive performance of five different GS methods for traits measured in maize inbred lines, and found that these methods differ slightly in their predictive abilities. Howard *et al.* (2014) compared the predictive abilities of parametric methods with nonparametric models using simulation data, and observed that parametric methods performed slightly better than nonparametric methods for predicting traits with more additive genetic components in their genetic architectures. However, nonparametric methods perform better than parametric methods when epistatic effects exists in a population (Howard *et al.* 2014).

Recently, several GS studies have paid closer attention to the fact that crop breeding lines are often assessed in multi environment trials (METs), *i.e.*, in different geographic locations, seasons, or years, in order to determine performance stability across environments (*i.e.*, G×E effects) (Pérez and de los Campos 2014; Crossa *et al.* 2016; Velu *et al.* 2016; Sukumaran *et al.* 2017). METs in a GS context are therefore an important extension as they allow the examination of marker by environment (M×E) interactions, and, in particular, the identification of markers whose effects are stable across environments, as well as those that are environment-specific (Crossa *et al.* 2016; Oakey *et al.* 2016). For example, López-Cruz *et al.* (2015) extended the single-trait, single-environment genomic estimated best linear unbiased prediction (GBLUP) model to a multi-environment context, and reported important gains in prediction accuracy with the multi-environment model relative to single-environment analysis in wheat. Cuevas *et al.* (2017) considered modeling G×E using both genetic markers and environmental covariates. These studies showed that modeling M×E interactions can give substantial gains in the prediction accuracy of GS.

The development of a new training population for GS requires considerable monetary and time inputs. Therefore, within a breeding program, well phenotyped historical populations, such as advanced breeding lines tested under numerous METs, may be suitable for implementation of GS in a commercial breeding setting. For example, Rutkoski *et al.* (2015) empirically demonstrated the utility of using historical wheat lines from several MET as a training population, although a training population of closer relatives increased predictive ability. Edriss *et al.* (2017) investigated GS accuracy using historical maize yield data from 2022 breeding lines tested across 156 trials in 18 different locations in Kenya, Tanzania, Ethiopia and Uganda.

The Commonwealth Scientific and Industrial Research Organization (CSIRO) cotton (*Gossypium hirsutum*) breeding program in Australia has been developing accessions targeting lint yield and fiber quality traits for the past 50 years. Most of these traits are complex and behave as quantitatively inherited traits (Campbell and Myers 2015). The polygenic architecture of the majority of these economically important traits make them extremely difficult to manipulate and improve. GS Bayesian models have proven to be advantageous for complex traits in other crops, such as grain yield where many loci of small effects control the trait (*e.g.*, Crossa *et al.* 2017). These traits are also expensive to measure and require relatively large amounts of seeds. For example, to obtain an accurate measurement of the most important trait, yield, requires significant replication both within a field, across geographical regions and over multiple seasons (Stiller and Wilson 2014). As a result, a complete breeding cycle, from initial crossing to commercial release, takes a minimum of eight to ten years.

In this context, we have compared the accuracies of different GS prediction models on two important end-use quality traits (fiber length and strength) that are regularly assessed by CSIRO’s cotton breeding program. The specific objectives of this study were (1) to evaluate the accuracy of GS for fiber length and strength, and (2) assess the merits of multi- over single-environment GS models in a practical breeding and selection context in cotton, a crop for which this has not previously been evaluated. GS models for cotton lint yield and lint percent, both much more genetically complex traits than fiber quality traits, are the subject of a future study. The long-term goal of this research is to optimize GS approaches and use it in cotton breeding to fast track the release of improved cotton varieties.

## Materials and methods

### Traits measured and structure of phenotypic data

In order to assemble a training population, we mined historical breeding lines that had both phenotype data (trait means) and remnant seeds for DNA extraction. These lines represented a mix of released cultivars, breeding lines and other external breeding lines developed and collected in an Australian cotton germplasm repository for the CSIRO cotton breeding program. The phenotype data used in this study were from breeding trials conducted from 1993 to 2010 at 7 sites (Table 1) and were not completely orthogonal (see Supplemental material File S2). The number of breeding lines that had seed available per site ranged from 80 to 215 (Table 1). Of the 7 sites, only four had an average of 116 breeding lines overlapping between sites and were used to test for marker stability across sites. The traits analyzed for the study were fiber length and strength. These traits were phenotyped using the Uster High Volume Instrument (HVI 900) as described in Liu *et al.* (2011) and Clement *et al.* (2012).

**Table 1:** Details of test sites for fiber quality traits over several years and estimates of genomic heritability (h^2^_g_) ± SEs

Site	Region^§^	Latitude	Longitude	Years	No. lines	h^2^_g_ ± SE. Fiber length	h^2^_g_ ± SE. Fiber strength
*Myall Vale (MV)	Central	30° 14’S	149° 38’E	1998-2004	215	0.62 ± 0.11	0.21 ± 0.10
Collarenebri (CO)	Central	29° 30’S	148° 44’E	1994-2002	88	0.33 ± 0.12	0.29 ± 0.11
*Bourke (BK)	Hot	30° 02’S	145° 57’E	1995-2010	125	0.51 ± 0.10	0.25 ± 0.13
*Emerald (EM)	Hot	23° 31’S	148° 10’E	1993-2005	124	0.52 ± 0.15	0.47 ± 0.08
*St. George (SG)	Hot	28° 08’S	148° 41’E	1993-2010	128	0.32 ± 0.14	0.29 ± 0.11
Breeza (BR)	Cool	31° 06’S	150° 31’E	1993-2005	80	0.42 ± 0.19	0.33 ± 0.13
Darling Downs (DD)	Cool	27° 22’S	150° 31’E	1993-2005	99	0.37 ± 0.13	0.42 ± 0.17

* Trials with 116 breeding lines in common and used for across-site and marker-by-environment (M×E) interaction GS models.

§ Region refers to Australian cotton belt which is divided into three regions, *i.e.*, hot, central and cool based on day-degrees (McMahon and Low 1972).

The trials were established in the Australian cotton belt which follows inland rivers of New South Wales and Queensland (http://cottonaustralia.com.au/uploads/publications/POCKET_GUIDE_MAP.pdf). The cotton belt is divided into three regions, *i.e.*, hot, central and cool based on day-degrees as described by McMahon and Low (1972). Day-degrees decline from North to South in the cotton cropping season (September to March) (Constable *et al.* 2001). Rainfall tends to be summer-dominant in the North and Central but winter-dominant in the South. On average, there are 248, 206 and 186 mm of in-crop rainfall for the central, hot and cool region, respectively (Liu *et al.* 2013) and more than 4700 MJ/m^2^ in radiation during the crop season (Rosenthal and Gerik 1991).

### Phenotypic data analysis

Phenotypic data were analyzed in a two-stage model. First, a within trial single site analysis was carried out using ASReml (Gilmour *et al.* 2009). The final adjusted means over site, trials, years and row-column designs were estimated with a mixed model applied to the alpha-lattice design of each trial using ASReml R (Butler *et al.* 2009; Gilmour *et al.* 2009). In order to minimize information loss when adjusted means are passed on from the first to the second stage analysis, the adjusted means were weighted by the inverse of their squared standard errors (*e.g.*, Cullis *et al.* 1996). The adjusted means for the breeding lines were then used in the second stage to predict GEBVs based on markers and corresponding marker effects.

### DNA extraction, SNP genotyping and calling

SNP genotyping was done using DNA isolated from cotyledons of 10-12 young seedlings with two true leaves of each accession. DNA extraction was performed using the DNeasy Plant Mini Kit (Qiagen) according to the manufacturer’s instructions. All DNA samples were quantified using a NanoDrop 1000 (Thermo Scientific) and normalized to the same concentration (Zhu *et al.* 2016).

DNA at 50 ng/µL for each of the breeding lines was processed according to Illumina protocols and hybridized to the CottonSNP63K array at CSIRO Agriculture and Food (Brisbane, Australia) according to the manufacturer’s instructions. Single-base extension was performed and the chips were scanned using the Illumina iScan. Image files were saved and analyzed using the GenomeStudio Genotyping Module (v 1.9.4, Illumina). Genotype calls for each SNP were performed based on the cluster file generated specifically for the CottonSNP63K array (Hulse-Kemp *et al.* 2015). The SNP calling module was developed for diploids, so for each locus there are three possible genotypes - *AA*, *AB*, and *BB*. Filtering was performed to return polymorphic SNPs with call rate above 80% and minor allele frequency > 5%. Missing data (4.8% of data points) were replaced by the mean value of the non-missing data for the loci, using the “mean” option implemented in the ridge regression best linear unbiased predictions (rrBLUP) package in R (Endelman 2011). A set of 13,330 polymorphic SNPs were used for model training and genomic predictions (see Supplemental material File S1). These SNPs were distributed across all the 26 chromosomes of cotton with a density of ∼5.3 SNPs/Mbp.

### Genomic relationship and population structure assessment

A random set of 5,000 SNPs were used to estimate the genomic relationship matrix (**G**) of the breeding lines, following VanRaden (2008). To explore genetic population structure in the breeding lines, principal component analysis (PCA) was performed on **G** using the R function ‘prcomp’ (R Development Core Team 2014) with 5000 SNPs chosen at random. This resulted in a matrix of eigenvectors (with dimensions equal to the number of breeding lines) ordered by descending eigenvalues with the largest eigenvalue for PC1. We refer to these eigenvectors of **G** as principal component (PC), where the PC1 had the largest eigenvalue. The PCs were plotted and annotated with population composition of the breeding lines to investigate where PCA was able to cluster the various accession composition together. We tested the significance of population structure following Patterson *et al.* (2006).

### Genomic prediction models at single-site level

We compared five Bayesian regression models (BRR, BayesA, BayesB, BayesC and RKHS) in order to identify the best model for genomic selection for implementation in cotton breeding. All prediction models were tested using the R (R Development Core Team 2014) “BGLR” package (Pérez-Rodríguez and de los Campos 2014).

All Bayesian models used in this study can be written as

γ=μ+Xb+ε [1]

where *γ* is the trait value, *µ* is the population mean, ***X*** matrix of marker-centered and standardized genotypes (*i.e.*, each marker was centered by subtracting the mean and standardized by dividing by the sample standard deviation), ***b*** is the vector of marker effects, and *ε* is the vector of model residuals. Marker effects and model residuals were assumed to be independent of each other and both normally distributed. The sum of all allele effects is the GEBV of the breeding line. Centering implies that variances and covariances between genetic values are measured as deviations with respect to a center defined by the average genotype.

### Estimating genomic heritability of the traits

The genomic heritability (h^2^_g_) (the proportion of variance of a trait that can be explained (in the population) by a linear regression on a set of markers (de los Campos *et al.* 2015b)) of each trait at each site was estimated from the genomic data. The genomic relationship matrix showed that our population is highly related and presumably comes from a very small effective population size as is typical of most cotton breeding lines (*e.g.*, Hinze *et al.* 2015; Gapare *et al.* 2017). Under these conditions a likelihood, constructed based on proportions of allele sharing at markers, is unlikely to be misspecified and consistency may hold, leading to very small bias in genomic heritability estimate. h^2^_g_ was estimated as the ratio of the genomic over the phenotypic variance, where the genomic variance is obtained with a REML analysis using the genomic relationship matrix (de los Campos *et al.* 2015b).

### Statistical models in genomic prediction at multi-site level

To assess the merits of multi- over single-environment GS models in cotton, we used phenotype data (adjusted means) from four sites that had 116 breeding lines in common (Table 1) and 13,316 SNPs. For comparison purposes, we performed a combined analysis based on an M×E model from which within-site analysis (single-site model) and across-site analysis can be derived and computed. All models were fitted using BGLR software – release 1.0.4; (de los Campos and Pérez-Rodríguez, 2015a) which support heterogeneous variances in the model residuals. We also applied BayesB that allows for incorporating priors that can induce variable selection and shrinkage simultaneously (Meuwissen *et al.* 2001) and also allows for environment-specific error variances (Crossa *et al.* 2016). The model is an extension of the López-Cruz *et al.* (2015) model that assumes homogeneity of within-site error variance.

A single-site regression model in matrix notation (Equation [2]) is similar to Equation [1] but can be obtained by removing the main effects of the markers, ***b***_0_ = 0, such that

$$\left[\begin{array}{l} y_{1}\\ y_{2}\\ y_{3}\\ y_{4} \end{array}\right]=\left[\begin{array}{l} 1_{\mu 1}\\ 1_{u2}\\ 1_{u3}\\ 1_{\mu 4} \end{array}\right]+\left[\begin{array}{cccc} X_{1} & 0 & 0 & 0\\ 0 & X_{2} & 0 & 0\\ 0 & 0 & X_{3} & 0\\ 0 & 0 & 0 & X_{4} \end{array}\right]\left[\begin{array}{c} b_{1}\\ b_{2}\\ b_{3}\\ b_{4} \end{array}\right]+\left[\begin{array}{c} \varepsilon_{1}\\ \varepsilon_{2}\\ \varepsilon_{3}\\ \varepsilon_{4} \end{array}\right]$$ [2]

All assumptions as specified in Equation [1].

The across-site model (Equation [3]) based on a GS model where marker effects are assumed to be constant across sites (*i.e.*, ignoring M×E). The model assumes heterogeneous error variances but assumes that marker effects are the same across sites such that ***b***_1_ *=* ***b***_2 =_ ***b***_3 =_ ***b***_4;_ and because all 116 breeding lines are tested at each site, $\chi_{1}$ = $\chi_{2}$ = $\chi_{3}$ = $\chi_{4}.$ The regression equation becomes a multi-variate model with the following notation:

$$\left[\begin{array}{c} y_{1}\\ y_{2}\\ y_{3}\\ y_{4} \end{array}\right]=\left[\begin{array}{c} 1_{\mu 1}\\ 1_{u2}\\ 1_{u3}\\ 1_{\mu 4} \end{array}\right]+\left[\begin{array}{c} \chi_{1}\\ \chi_{2}\\ \chi_{3}\\ \chi_{4} \end{array}\right]b+\left[\begin{array}{c} \varepsilon_{1}\\ \varepsilon_{2}\\ \varepsilon_{3}\\ \varepsilon_{4} \end{array}\right]$$ [3]

A marker×environment model (M×E) (Equation [4]) allows analyzing data from all sites jointly and accounts for G×E. In M×E model, the effect of the *k*th marker in the *j*th environment is modeled as the sum of a main effect (***b****_0k_*) plus an interaction term ***b****_jk_* representing deviations from the main effect resulting from M×E. The marker effect model for the *k*th marker in the *j*th environment is ***b****_jk_* = ***b****_0k_* + ***b****_jk_*. The model can be expressed in matrix notation and for four environments as:

$$\left[\begin{array}{c} y_{1}\\ y_{2}\\ y_{3}\\ y_{4} \end{array}\right]=\left[\begin{array}{c} 1_{\mu 1}\\ 1_{u2}\\ 1_{u3}\\ 1_{\mu 4} \end{array}\right]+\left[\begin{array}{c} \chi_{1}\\ \chi_{2}\\ \chi_{3}\\ \chi_{4} \end{array}\right]b_{0}+\left[\begin{array}{cccc} X_{1} & 0 & 0 & 0\\ 0 & X_{2} & 0 & 0\\ 0 & 0 & X_{3} & 0\\ 0 & 0 & 0 & X_{4} \end{array}\right]\left[\begin{array}{c} b_{1}\\ b_{2}\\ b_{3}\\ b_{4} \end{array}\right]+\left[\begin{array}{c} \varepsilon_{1}\\ \varepsilon_{2}\\ \varepsilon_{3}\\ \varepsilon_{4} \end{array}\right]$$ [4]

where the vectors of the main and interaction effects and model residuals were all assumed to be normally distributed, specifically ***b****_0_* ∼ *N*(0,*Iσ^2^_b0_*), ***b****_j_* ∼ *N*(0,*Iσ^2^_bj_*) and ***ε*** *∼N*(0,**D** ⊗ **I**_n_), where **D** = diag (*σ^2^_ε1_*_,_ *σ^2^_ε2_*, *σ^2^_ε3_*_,_ *σ^2^_ε4_*.) denoting the residual variance for each site, and **I**_n_ is an *n*-dimensional identity matrix.

All models were adjusted via Bayesian approach, using Gibbs Sampler MCMC (Markov chain Monte Carlo) method. Thus, we obtained marginal posterior distributions equal to the restricted maximum likelihood (REML) procedure, to obtain variance and covariance components, and to compute the GBLUP values. In the implementation of the Gibbs Sampler, we considered 210,000 samples collected from the posterior distribution after discarding 10,000 for burn-in, and considering 20 as thinning.

### Estimating accuracy of genomic predictions

To assess GS prediction accuracy at each site, we generated multiple TRN–TST partitions with random assignment of breeding lines to either TRN (training) or TST (testing) subsets of the whole dataset. This approach was favored because with a replicated TRN-TST design, one can obtain as many partitions as one needs and this allows estimating standard errors of prediction accuracy more precisely than with a cross-validation approach (*e.g.*, López-Cruz *et al.* 2015). For assessment of prediction accuracy across-sites, we adapted cross-validation (CV) schemes of Burgueño *et al.* (2012): first CV1 that mimics the prediction problem faced by breeders when breeding lines have not been evaluated in any field trials (López-Cruz *et al.* 2015); second CV2 that evaluates the prediction ability of models when some breeding lines have been evaluated in some sites, but not in others (López-Cruz *et al.* 2015). We used an R code provided in López-Cruz *et al.* (2015) supporting information to generate TRN-TST partitions in CV2. This validation mimics the prediction challenge faced by breeders where breeding lines are evaluated in some, but not all target environments across an industry (López-Cruz *et al.* 2015). In such an evaluation, information from related breeding lines and the correlated sites is used, and prediction assessment benefits from borrowing information between breeding lines within a site, between breeding lines across sites, and among correlated sites (Burgueño *et al.* 2012).

For both single-site and across-sites predictions, the data were divided randomly into 50 partitions, with 70% of the breeding lines assigned to the TRN set used to derive the models and 30% assigned to the TST set to test those models. The adjusted means of breeding lines at each site were used as observed phenotypic records in the GS model. Each partition yielded a point estimate of prediction accuracy (*e.g.*, correlation coefficient between predictions and adjusted phenotypes). The variability of the point estimate across partitions (replicates) reflects uncertainty due to sampling of TRN and TST sets, and a precise estimate of prediction accuracy was obtained by averaging the estimates of accuracy obtained in each partition. In selection theory, the accuracy is defined as the correlation between the selection criterion and the true breeding value (TBV). As calculated here, this correlation is reduced by deviations of the phenotype from the TBV. The average correlation for each trait was divided by the square root of the heritability of the respective trait (*e.g.*, Lorenzana and Bernardo 2009; Daetwyler *et al.* 2013). For each model-trait combination, we also evaluated the bias associated with the prediction. The slope coefficient (***b***) for the linear regression of GEBVs of the validation set on their EBVs was defined as a measurement of the degree of bias of a model. In this case, ***b*** ≥ 1 indicates no bias, whereas ***b*** < 1 indicates a biased overestimation of GEBVs.

## Data Availability

Genotype data and Phenotype data are included in Supplemental materials File S1 and File S2, respectively. The authors confirm that all data necessary for confirming the conclusions are presented fully in this article.

## Results

### Phenotype data

Genotype by year interaction accounted for only 1.14% and < 0.5% of the total phenotypic variation for fiber length and strength, respectively. Boxplots of standardized cotton fiber length and strength showed that the overall empirical distribution of each trait was approximately normal (data not shown), allowing the use of Gaussian distribution models. Genomic heritability estimates at each site for each traits are presented in Table 1 (see materials and methods section). Individual site estimates were varied for both traits, ranging from 0.21 to 0.62. The estimates across-sites averaged 0.44 and 0.32 for fiber length and strength, respectively.

### Genomic relationship and population structure

**Figure 1:**
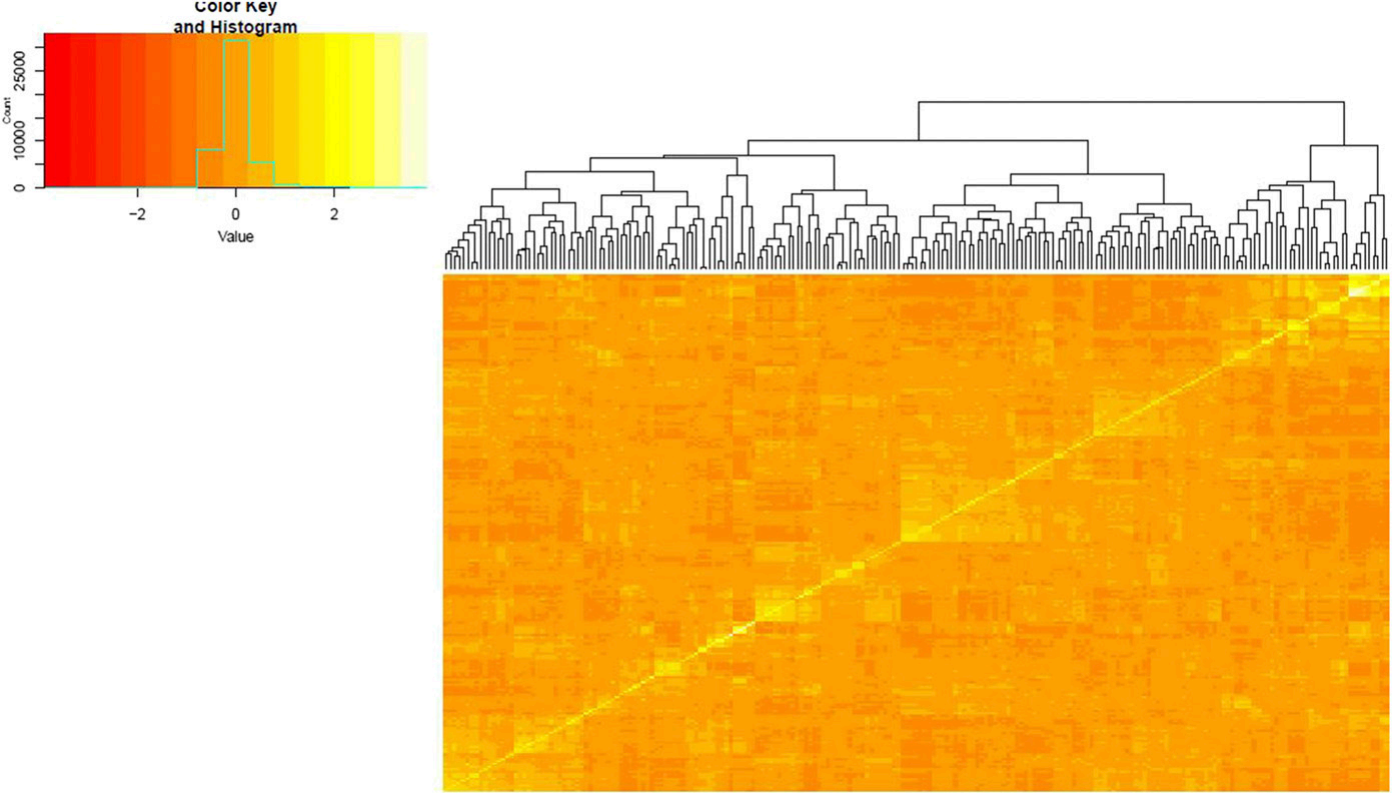
Heat map of the G matrix of 215 cotton historical breeding lines genotyped with 13,330 SNP markers.

**Figure 2:**
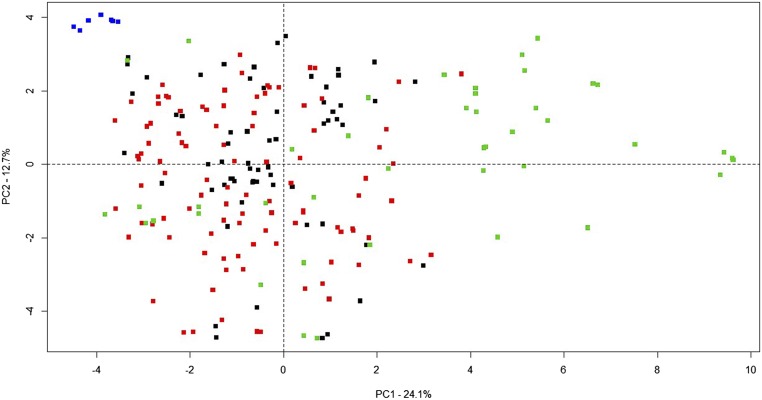
Plot of principle component (PC) 1 *vs.* PC 2 scores for each historical breeding line (*N* = 215). Principal component analysis performed on genomic relationship matrix (G) estimated from single nucleotide polymorphism data for each breeding line. Green, red, black and blue squares represent a mix of elite varieties and overseas introduced lines, lines derived from pre-2000, post-2000 crosses and elite varieties, respectively.

Figure 1 depicts the heatmap of the genomic (**G**) matrix for 215 breeding lines. The 215 breeding lines comprise three groups, one small group (top left) with two subgroups unrelated to one either of the two and two other large groups, each with a number of subgroups closely related to each other. Principal component analysis was performed to gain insight into population structure that may affect prediction accuracy (Figure 2). The first axis (PC1), which explained ∼24.1% of the variability present in the genomic data, showed no obvious clustering but evidence of strong admixture of breeding lines from pre-2000 crosses and post-2000 crosses. There was also evidence of wide dispersion of a limited number of breeding lines especially defined by PC1. We accepted the hypothesis of absence of significant population structure using *P* = 0.01. Thus, we did not detect distinct genetic groups in this population but instead evidence of shared common alleles as seen by the mixture of colors representing different breeding lines in the biplot.

### Model selection using data From Myall Vale

We tested all the five models using data from each site and they gave similar results, so we only present results from one site (Myall Vale) that had the most number of breeding lines. Table 2 provides five different commonly used models for GS and their respective residual variance estimates and the deviance information criterion (*DIC*) (measures of goodness of fit and model complexity) for each of the traits tested at Myall Vale site using 215 breeding lines. The five models gave very similar estimates and predictions, as the correlation between phenotypes and GEBVs derived from each of the models were relatively high (Table 2). However, a smaller *DIC* is preferable suggesting that BayesB was the preferred model for both traits. The model with the smallest *DIC* also had the minimum residual variance. The phenotypes were standardized to a unit sample variance and the estimated residual variances for fiber length was 0.233, thus the model explained ∼77% of its phenotypic variance. The preferred model for fiber strength only explained 29% of the phenotypic variance. Based on residual variance estimates, all models fitted the data better for fiber length than fiber strength (Table 2).

**Table 2:** Measures of goodness of fit for different models for two fiber quality traits using data at Myall Vale site

Trait		Models				
		BRR	BayesA	BayesB	BayesC	RKHS
Fiber length	Res var (SD)	0.244 (0.05)	0.236 (0.04)	0.233 (0.05)	0.243 (0.05)	0.242 (0.05)
	*DIC*	402.86	398.99	**396.88**	402.21	401.7
	PA	0.96	0.96	0.96	0.95	0.94
Fiber strength	Res var (SD)	0.727 (0.09)	0.708 (0.09)	0.706 (0.09)	0.723 (0.10)	0.745 (0.09)
	*DIC*	587.46	587.15	**586.27**	587.00	592.88
	PA	0.78	0.81	0.82	0.79	0.77

Res var = residual variance; BRR = Bayesian Ridge Regression; RKHS = Reproducing Kernel Herbert Spaces Regression; SD = Standard Deviation; *DIC* = Deviance Information Criterion; *DIC* in bold was the best model for the trait; PA = Prediction Accuracy – *i.e.*, correction between phenotypes and genomic estimated breeding values.

### Estimates of variance components From Across-site and M×E models

Table 3 provides phenotypic correlations of fiber length and strength across four sites that had common breeding lines. Correlations among sites were all positively correlated. For fiber length, correlations ranged from 0.51 to 0.68 and were higher for strength, ranging from 0.71 to 0.81.

**Table 3:** Sample phenotypic correlation estimates ± SE for fiber length and strength evaluated at four sites

Trait -Length	Emerald	St George	Myall Vale
Bourke	0.68 ± 0.07	0.63 ± 0.07	0.60 ± 0.07
Emerald		0.52 ± 0.08	0.68 ± 0.06
St George			0.51 ± 0.09
Trait -Strength			
Bourke	0.71 ± 0.07	0.76 ± 0.06	0.79 ± 0.06
Emerald		0.81 ± 0.05	0.80 ± 0.06
St George			0.77 ± 0.06

Estimates of residual and genomic variance components for the single-site, across-site and M×E models are presented in Table 4. For all traits and sites, the estimated residual variances for the M×E model were always smaller than those derived from the single-site and across-site models. These results indicate the M×E model fits the data better than models that (a) force the marker effects to be constant across sites and (b) single-site models.

**Table 4:** Estimated posterior residual variance components (and their posterior standard deviations, SD) and the estimated posterior probability of markers with nonnull effects from the single-site, across-site and the marker × environment interaction models for fiber length (LEN) and strength measured at four sites

		Fiber length		Fiber strength	
	Site	Estimate	SD	Estimate	SD
		**Single-site**			
Residual	Myall Vale	0.469	0.13	0.405	0.12
	Bourke	0.407	0.13	0.446	0.13
	Emerald	0.401	0.13	0.463	0.13
	St George	0.465	0.12	0.488	0.12
Probability	Myall Vale	0.321	0.15	0.158	0.06
	Bourke	0.385	0.18	0.219	0.09
	Emerald	0.377	0.21	0.214	0.10
	St George	0.301	0.17	0.182	0.07
		**Across-site**			
Residual	Myall Vale	0.458	0.09	0.298	0.07
	Bourke	0.309	0.06	0.310	0.06
	Emerald	0.289	0.06	0.260	0.06
	St George	0.750	0.13	0.269	0.07
Probability	All	0.298	011	0.238	0.06
		**M×E**			
Residual	Myall Vale	0.341	0.06	0.225	0.05
	Bourke	0.243	0.04	0.264	0.05
	Emerald	0.254	0.05	0.209	0.04
	St George	0.619	0.11	0.213	0.04
Probability	Main effect	0.382	0.07	0.376	0.07
environment	Myall Vale	0.504	0.08	0.462	0.08
main effect and	Bourke	0.216	0.08	0.200	0.07
specific effect	Emerald	0.435	0.12	0.444	0.16
	St George	0.567	0.09	0.500	0.10

For the M×E interaction model (BayesB), marker effects are given in terms of the probability or proportion of markers with effects different from zero (nonnull) that are estimated for each of the components of the marker effects (*i.e.*, main effect and site marker specific effect (see Table 4). Overall, across-site model had a lower proportion of markers with nunnull effect than the M×E model (Table 4). For example, across-site model gave proportions of 29.8 and 23.8% of markers with nunnull effects for fiber length and strength, respectively, whereas the M×E model gave, for the marker main effect, proportions of 38.2 and 37.6% of the markers with nunnull effects for fiber length and strength, respectively. On average, the environment-specific proportion of markers with nunnull effect were greater than main effect, with the exception of Bourke (Table 4).

### Prediction accuracy based on multiple training-testing partitions at each site

**Figure 3:**
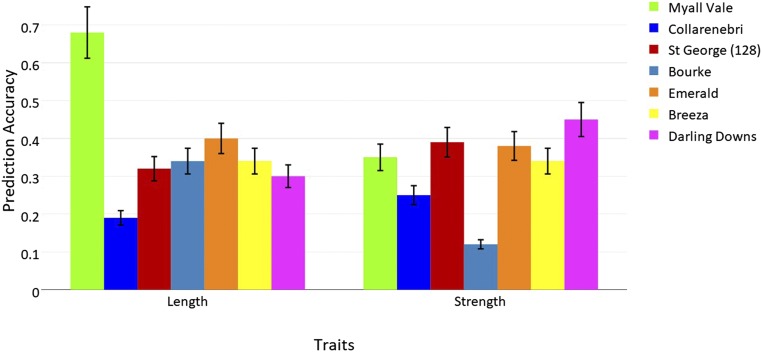
Estimated prediction accuracy (prediction accuracy between phenotypes and predictions averaged over 50 TRN-TST partitions) for cotton fiber length and fiber strength at seven test sites.

Prediction accuracy based on training-testing partitions (TRN70-TST30) for fiber length and fiber strength at each of the seven sites individually are shown in Figure 3. Prediction accuracy for fiber length ranged from 0.27 at Collarenebri to 0.77 at Myall Vale (with the highest number of breeding lines). However, at five of seven sites, excluding Collarenebri, prediction accuracy averaged 0.46. For fiber strength, accuracies ranged from 0.19 at Bourke to 0.58 at Darling Downs, with an average of 0.50, excluding Bourke.

### Assessment of prediction accuracy in a multi-site context for fiber length and strength

Prediction accuracies obtained under different models for fiber length and strength are presented in Table 5 (CV1) and 6 (CV2). Single-site analysis by either validation approaches yielded moderately low prediction correlations for fiber length, ranging from as low as 0.19 for to 0.34 (Tables 5 and 6). Similarly, low prediction correlations were observed for fiber strength, ranging from 0.12 to 0.35 (Tables 5 and 6). Under CV1, across-site model and the M×E model performed similarly to single-site model. For an across-site model that analyzed data jointly under CV2, the model yielded higher prediction accuracies than single-site models for length and strength, with an average prediction correlation of 0.41 and 0.39, respectively (Table 6). Prediction accuracies from the M×E model were higher than those for the across-site model, with an average prediction accuracy of 0.71 and 0.59 for fiber length and strength, respectively (Table 6). For all models and trait combination, the slope coefficients were not significantly different than 1, *i.e.*, indicating no significant bias in the GEBVs prediction.

**Table 5:** Estimated prediction accuracy (correlation coefficient between predicted and observed phenotypes, averaged over 50 TRN-TST partitions) ± SE for fiber length and strength in cotton by CV1

Trait/Sites	Prediction accuracy ± SE			
Fiber length	Single site	Across-site	M×E model	Selection efficiency (%)*^a^*
Myall Vale	0.19 ± 0.02	0.14 ± 0.02	0.16 ± 0.03	27; 37
Bourke	0.23 ± 0.03	0.23 ± 0.02	0.23 ± 0.02	24; 38
Emerald	0.33 ± 0.02	0.26 ± 0.03	0.29 ± 0.02	23; 37
St George	0.30 ± 0.02	0.22 ± 0.02	0.28 ± 0.02	19; 37
Mean	0.26	0.21	0.24	23; 37
Fiber strength				
Myall Vale	0.26 ± 0.04	0.19 ± 0.04	0.19 ± 0.03	35; 52
Bourke	0.14 ± 0.02	0.13 ± 0.04	0.11 ± 0.02	28; 39
Emerald	0.35 ± 0.05	0.28 ± 0.02	0.29 ± 0.02	42; 51
St George	0.26 ± 0.02	0.26 ± 0.03	0.26 ± 0.03	43; 54
Mean	0.25	0.22	0.14	37; 49

a Selection efficiency across-site model relative to single-site (before semi-colon) and relative to M×E model (after semi-colon).

**Table 6:** Estimated prediction accuracy (correlation coefficient between predicted and observed phenotypes, averaged over 50 TRN-TST partitions) ± SE for studied traits in cotton, CV2

Trait/Sites	Prediction accuracy ± SE			
Fiber length	Single site	Across-site	M×E model	Selection efficiency (%)*^a^*
Myall Vale	0.19 ± 0.02	0.38 ± 0.02	0.64 ± 0.14	31; 39
Bourke	0.21 ± 0.02	0.33 ± 0.09	0.41 ± 0.11	28; 41
Emerald	0.34 ± 0.02	0.51 ± 0.03	0.65 ± 0.13	21; 23
St George	0.33 ± 0.02	0.42 ± 0.02	0.63 ± 0.14	33; 38
Mean	0.27	0.41	0.71	28; 35
				
Fiber strength				
Myall Vale	0.25 ± 0.01	0.31 ± 0.09	0.52 ± 0.11	32; 47
Bourke	0.12 ± 0.02	0.28 ± 0.11	0.57 ± 0.11	29; 36
Emerald	0.34 ± 0.02	0.48 ± 0.12	0.61 ± 0.14	48; 57
St George	0.34 ± 0.02	0.49 ± 0.15	0.64 ± 0.13	49; 52
Mean	0.26	0.39	0.59	40; 48

a Selection efficiency across-site model relative to single-site (before semi-colon) and relative to M×E model (after semi-colon).

To compare changes in ranking of the breeding lines based on GEBVs from each of the models, we used the coincidence index, which estimates the selection efficiency of multi-site relative to single-site models (*e.g.*, Hamblin and Zimmermann, 1986 – page 253). Briefly, when the number of breeding lines identified by the multi-site and M×E interaction models is no more than the number of breeding lines expected by chance, the selection efficiency is zero (*e.g.*, Hamblin and Zimmermann 1986). Likewise, when the number of breeding lines identified in the multi-site and M×E interaction models is the same as the number selected by the single-site model, the selection efficiency is 100%. Each site had 116 breeding lines and therefore a selection intensity of 20% identified 23 breeding lines. Assuming a random relationship of the breeding lines in different models, approximately 5 breeding lines (23 × 20%) would be identified by chance. For example, under CV1 at Myall Vale, 5 of the breeding lines identified using the single-site model were also in the top 20% identified by the multi-site model. The selection efficiency in this case would be estimated as follows:

Selection  efficiency = [(10-5) /(23-5)]×100=28%

Under CV2, mean selection efficiency of the across-site and M×E interaction models over single-site model was 40% and 48% for fiber length and strength, respectively (Table 6).

## Discussion

Genomic selection is now taking advantage of the large amount of phenotypic data collected by breeding programs across years, provided there is seed available DNA extraction upon which we get genotypic data. However, this also raises new challenges to optimally exploit those data. By nature, historical data can be extremely unbalanced. Historical data will include trials of varying quality without readily accessible meta-information on issues affecting trial quality (*e.g.*, Heslot *et al.* 2013; Rutkoski *et al.* 2015). This study demonstrated that historical data on fiber length and strength when combined with high density SNP data, can be used to develop predictive models for complex agronomic traits of cotton. Varying training population sizes ranging from 200 to 500 individuals were used in most initial GS studies in other crops (*e.g.*, Lorenzana and Bernardo 2009; Crossa *et al.* 2014; Spindel *et al.* 2015). Some studies have reported genetic gain to double even when using merely 500 individuals, compared to phenotypic selection. For example, Lorenz (2013) and Lin *et al.* (2016) have found that GS with optimized breeding designs can enhance genetic gain, while consuming less cost per unit time as compared to traditional breeding.

This study focused on two economically important fiber quality traits – fiber length and strength that have similar and reasonably moderate genomic heritabilities. We were not able to compare our genomic heritability estimates from other studies as there are no estimates in the literature, except for those estimated using the additive relationship matrix. However, our estimates fell within the range reported by Campbell and Myers (2015) that showed that the estimates range from 0.23 to 0.57 for both traits, depending on the population sampled and the environment for which they were evaluated. Because of a high level of relatedness in the breeding lines, they likely share long chromosome segments and, under these circumstances, the patterns of allele sharing at markers and at QTL are very similar. This leads to very small bias in genomic heritability estimates (*e.g.*, de los Campos *et al.* 2015b).

Heritability of a trait corresponds to the upper limit of the phenotypic variance explained by a linear genetic prediction model (Clark *et al.* 2012; Wray *et al.* 2013). A linear relationship was expected, since traits with moderate heritability, normally, present phenotypes with expression of relatively high genetic variance, and are expected to be more predictable by a genomic approach. This was the case for fiber length at most sites but uniquely not fiber strength at Myall Vale, where the residual variance was almost 70%, suggesting that other factors not accounted for by the model were contributing to phenotypic variance (Table 2). Incorporating other factors such as environmental covariates would reduce the amount of residual variance for fiber strength (*e.g.*, Jarquín *et al.* 2014).

### Model selection

Complex traits are possibly affected by large numbers of small-effect QTL and the analysis of such traits requires fitting a large number of variants concurrently using a GS approach such as the one proposed by Meuwissen *et al.* (2001). Since the introduction of genomic selection models, empirical evidence has demonstrated that no single model performs best across species and traits and grouping traits based on their architecture is not always straightforward (Heslot *et al.* 2012; de los Campos *et al.* 2013). All five models performed similarly although, the deviance information criterion favored BayesB which is based on specifying all SNP-associated effects to be independent of each other, allowing a large proportion of SNP markers to be associated with null effects (Meuwissen *et al.* 2001; Yang and Tempelman 2012). When markers and QTL co-segregate, variable selection does not seem to be needed (de los Campos *et al.* 2015b). Given that fiber length and strength are moderately heritable, we would expect both traits to be associated with many loci, each explaining only a small portion of the genetic variance. We would expect BayesB to have increased prediction accuracy over BRR or RR-BLUP because they avoid over-shrinking those QTL with moderate effects (*e.g.*, de los Campos *et al.* 2013). We would also expect prediction accuracies from BayesB to persist in new populations because they focus more on analyzing QTL-marker associations rather than on relationships, whereas RR-BLUP rely on kinship more strongly. The genetic correlation between the two traits is known to be positive and high ∼0.80 (*e.g.*, Lacape *et al.* 2005; Gapare *et al.* 2017). Likewise, we would expect the two traits to be controlled by similar sets of loci, although the influence of other factors on fiber strength cannot be excluded, given that the residual variance for fiber strength was much higher than that for fiber length.

Marker density plays an important role in genomic prediction. For GS, it is desirable to obtain adequate genome coverage so that all contributing QTL are in LD with at least one marker. Studies have shown this optimum marker size to be trait and population specific (Heffner *et al.* 2011). The SNPs used in our study were distributed across all the 26 chromosomes of cotton with a density of ∼5.3 SNPs/Mbp. Implementing GS into a breeding program would likely involve increasing training panel size over time as more phenotypic data are generated. Thus, as more individuals are added to the model, there may be a need to reassess the optimum marker number. However, if the accuracy obtained in this study is due to LD, then it is more likely to persist across generations and breeding lines than if the accuracy due to relationships (*e.g.*, Habier *et al.* 2007; Habier *et al*. 2013).

### Relationships and population structure

Naturally and artificially selected/breeding populations usually exhibit some degree of relatedness and stratification. Pronounced population structure has to be considered when evaluating the potential of genomic selection (Isidro *et al.* 2015). In general, when population structure is not taken into account, genomic prediction accuracy decreases (Sallam *et al.* 2015). As might be expected for breeding lines that have gone through repeated selection and breeding, leading to relatedness. The heat map based on **G** matrix suggested that our population was highly related. In our study, we did not detect any evidence of significant subpopulation structure although, there was evidence of strong admixture, with 24.1% of the total variation being explained by PC1 and 12.7% by PC2.

### Prediction accuracy based on multiple training-testing partitions at each site

Prediction accuracy for fiber strength at Myall Vale was almost half that observed for fiber length. However, based on their known heritabilities, we had expected that prediction accuracy for fiber strength would be in the same range as fiber length. Possible reasons for such low prediction accuracy for strength include (i) model inadequacy as evidenced by the high residual variance (Table 2); (ii) the genomic region affecting the trait might not have been effectively covered in the current genotyping data using a SNP chip that does have some gaps in coverage on some chromosomes (Hulse-Kemp *et al.* 2015), and (iii) small size of the training population. There are ongoing efforts to increase the size of the training population. Accuracy may improve when we can move to more extensive genotype-by-sequencing approaches to genotyping. One possibility would be to expand any of the previously presented models by including an interaction term between environmental covariates and the random effect of the markers. For example, LD between markers and genes at causal loci or because of model misspecification (*e.g.*, interactions between alleles that are unaccounted for), the regression on markers may not fully describe genetic differences among lines (*e.g.*, Jarquín *et al.* 2014).

With the exception of Myall Vale, prediction accuracies for fiber length at each site were almost the same, averaging 0.43. One possible reason for differences in prediction accuracy between Myall Vale and other sites may be the high genomic heritability estimate and also the size of the training population that was almost double that of any other site (Table 1). Some of the variation observed in our results could be due to other unmeasured features (*e.g.*, environmental variables), because accuracies from prediction models depend on a complex network of different, interrelated factors. The unknown factors may explain some proportion of the variation in the datasets and increase the power of the model for prediction.

### Variance components From Across-site and M×E models

Our results agreed with those found by López-Cruz *et al.* (2015) and Crossa *et al.* (2016), where the M×E model fitted the data better than the single-site and across-site models. In this study, fiber length and strength had positive and high sample phenotypic correlations among sites. The across-site model had relatively higher residual variance than the M×E model, indicating that forcing constant marker effects across sites, ***b***_1_ *=* ***b***_2_ = ***b***_3_ = ***b***_4_, does not produce a better fit of this model (*e.g.*, Crossa *et al.* 2016). The M×E model is based on variance component estimation of the marker main effects and site-specific marker effects and, in terms of prediction accuracy, it performed well at all sites since they were positively correlated (Table 3). As might be expected for traits that have positive correlations between sites, the M×E model will tend to have higher prediction accuracy for traits that have positive correlations than traits with zero or negative correlations between sites (*e.g.*, López-Cruz *et al.* 2015).

### Genomic selection accuracy Across sites

Genomic prediction models have been proposed that take into account the random effects of markers and their interaction with environments based on genetic and environmental similarities among individuals (Jarquín *et al.* 2014; Perez-Rodriguez *et al.* 2015; Cuevas *et al.* 2017). As might be expected for traits that are moderately influenced by environment, M×E model outperformed both single- and across-site models. Overall, the M×E model performed best under CV2 (Table 6). The low prediction accuracies under CV1 and higher accuracies under CV2 are consistent with those of previous studies in cotton (*e.g.*, Perez-Rodriguez *et al.* 2015) and in wheat (Burgueño *et al.* 2012; Lado *et al.* 2016) that have used similar cross-validation designs. These studies also found that the inclusion of G×E produced a considerable increase in prediction accuracy. Predictions in TST data sets are derived using information from the genotypes included in the TRN data; therefore, prediction accuracy depends on how much information can be borrowed for genotypes in the TRN set that is relevant to those in the TST set (López-Cruz *et al.* 2015; Velu *et al.* 2016; Sukumaran *et al.* 2017).

Our results support evidence of increased accuracies by incorporating G×E terms in prediction models (Table 6). For example Perez-Rodriguez *et al.* (2015) demonstrated that prediction accuracies in cotton yield could increase from 0.45 to 0.51 by using models that incorporate G×E terms. One advantage of the M×E model is that it can be used with priors that induce differential shrinkage of estimates as well as priors that produce variable selection (de los Campos *et al.* 2013). Such treatment would potentially aid in identifying sets of markers with effects that are stable across environments and others that are responsible for G×E (López-Cruz *et al.* 2015). For example, Crossa *et al.* (2016) used the M×E GS model to identify genomic regions in which the effects are stable across environments and other regions that are specific to certain environments and therefore responsible for G×E in grain yield in durum wheat. We envisage that this model would be useful for fiber yield components traits that exhibit considerable amount of G×E (*e.g.*, Perez-Rodriguez *et al.* 2015) and have low heritability (Liu *et al.* 2011).

Using data from one site to predict the performance in independent sites is crucial for plant breeding. The GS prediction accuracy between sites for fiber length and strength were moderately high (Table 6). The M×E model under CV2 is based on variance component estimation of the marker main effects and environment-specific marker effects and, in terms of prediction accuracy, it performed well at all sites. This is an important result for cotton breeding given that all initial selection and breeding are carried out at the breeding program base location (Myall Vale site) with the follow-up of elite lines being tested across regions in breeding target environments. Thus, as for fiber length and strength, the ability of the data from one environment to predict the other, was similar, irrespective of which site’s data were used to build the prediction model.

### Conclusions

GS is still in its infancy in most plant breeding programs, and one of the biggest obstacles for implementing GS in practical breeding is the high start-up costs required. The investment in starting GS is substantial in both technology and human resources required with regards to the costs of phenotyping, maintaining a large training population, costs of genotyping entire breeding populations and model optimization analysis. However, genotyping costs are continually decreasing and genotyping of large plant populations is much more manageable today than it was just a few years ago. We also envisage that further cost reductions could be made by utilizing historical phenotype data, as it would reduce the costs of establishing, phenotyping, and maintaining initial training populations significantly. If historical data can be correctly adjusted for annual variation of environmental factors, they represent a substantial resource. Historical data could be used to initiate a GS breeding program, allowing breeders to realize the potential and benefits of GS, before incorporating contemporary data and recalibrating the model.

Further work is required to increase the size of the training population and test the models on cotton fiber yield and other fiber quality traits before implementation of GS in cotton breeding. In the near future, a model that integrates the analysis of multi-traits and multi-environments and takes into account trait × genotype × environment interaction (T×G×E) in a unified whole genome prediction would be desirable. GS could have its greatest potential use at points in the breeding program where selection using traditional methods (for example, through the generation of phenotypes via replicated trials) is too expensive, time consuming, or not biologically or logistically possible because of the wide geographical distribution of the industry in Australia (∼1,500 km from hot to cool climates). The most important questions relating to the applicability of GS in the CSIRO cotton breeding program are whether it will better help breeders predict: (1) breeding values of individuals for more rapid selection cycling or (2) genotypic values of advanced lines that are in the last stages of testing and we believe that for the traits examined in this study that it will provide value if adopted as a routine part of Australian cotton breeding. This study can contribute to breeding programs for other crops, like wheat or barley, where a conventional strategy of selection based on the phenotype is used and much historic phenotype data are available that has been collected over multiple years by breeders.

## Supplementary Material

Supplemental Material is available online at www.g3journal.org/lookup/suppl/doi:10.1534/g3.118.200140/-/DC1.
